# Public Knowledge about and Detection of Canine Visceral Leishmaniasis in Urban Divinópolis, Brazil

**DOI:** 10.1155/2012/429586

**Published:** 2012-09-06

**Authors:** Carina Margonari, Júlia Alves Menezes, Marcele Neves Rocha, Kamila Nunes Maia, Michael Éder de Oliveira, Amanda Luisa Fonseca, Fabrizio Furtado de Sousa, Eduardo de Castro Ferreira, Ana Paula Madureira, Maria Norma Melo, Rodrigo Pedro Soares

**Affiliations:** ^1^Centro de Pesquisas René Rachou, Fundação Oswaldo Cruz/FIOCRUZ, Avenida Augusto de Lima 1715, 30190-002 Belo Horizonte, MG, Brazil; ^2^Fundação Educacional de Divinópolis, Universidade Estadual de Minas Gerais (FUNEDI/UEMG), Avenida Paraná 3001, 35501-170 Divinópolis, MG, Brazil; ^3^Departamento de Ciências Médicas, Escola de Farmácia, Universidade Federal de Ouro Preto, Rua Diogo de Vasconcelos 122, 35400-000 Ouro Preto, MG, Brazil; ^4^Departamento de Engenharia de Biossistemas, Universidade Federal de São João del-Rei, Praça Frei Orlando, 170 Centro, 36307-352 São João del-Rei, MG, Brazil; ^5^Departamento de Parasitologia, Universidade Federal de Minas Gerais, Avenida Antônio Carlos 6627, 31270-901 Belo Horizonte, MG, Brazil

## Abstract

*Background*. Leishmaniases are diseases with a wide spectrum of clinical manifestations including cutaneous (CL) and visceral (VL) forms. Many factors may affect their occurrence and expansion including environmental, geographic, and social conditions. In the past two decades, Divinópolis, Minas Gerais State, Brazil, has exhibited the potential for a disease outbreak, with the appearance of CL, and VL cases (human and canine). Hence, this study was initiated to monitor public knowledge of the disease. Questionnaires were administered in four neighborhoods (Jardim Belvedere, Esplanada, Danilo Passos I and II) where most of the human and canine cases have been reported. The analyses demonstrated that public knowledge of the disease is sparse and fragmented. A strong perception of the dog as the main reservoir was observed. Five veterinary clinics were evaluated for the presence of canine VL using serological (RIFI and ELISA) and molecular (PCR-RFLP) techniques. This is the first study demonstrating the occurrence of *Leishmania infantum* in Divinópolis, suggesting a possible urbanization of VL.

## 1. Introduction

Leishmaniases are a group of diseases caused by the protozoan *Leishmania *(Kinetoplastida: Trypanosomatidae) affecting 12 million people in 88 countries. The disease exhibits a wide spectrum of clinical manifestations ranging from benign cutaneous lesions (CL) to the fatal visceral form (VL) [1]. In Latin America, especially Brazil, both forms are widely distributed and are transmitted by the bite of phlebotomine sand flies (Diptera: Psychodidae) [2]. Wild and domestic reservoirs including foxes, marsupials, rodents, dogs, and cats are the main sources of sand fly infection [3–8].

Many factors may have contributed to VL and CL expansion and urbanization [9], including deforestation [10], human migration [11], vector adaptation [12, 13], drug resistance [14], poverty [15], and social conflicts [16]. As a result of anthropic modifications, VL has been increasingly reported in urban areas of major Brazilian cities including Natal, Teresina, Sobral, and Belo Horizonte [5, 17–20].

The city of Divinópolis, Minas Gerais State, Brazil, has a population of approximately 210,000. It has grown dramatically, with 90.5% of its territory completely urbanized in 2000 [21]. During the 1990s, 135 CL cases were detected by health authorities. Most of those cases were reported in the neighborhoods of Jardim Belvedere, Esplanada, São José, Catalão, and Candelária, all in the vicinity of Mata do Noé forest, where a large area was deforested. More recently, another forest remnant, Gafanhoto Park, was reported to be a potential CL focus, where known vectors and reservoirs were detected [22]. Between 2004 and 2008, 33 canine VL cases were detected, and this number increased up to 215 in 2010. This was also followed by an increase in the number of human VL and CL cases (5 and 16, resp.) for the same period (Table 1) [23, 24]. Those data were primarily based on notification by health professionals rather than due to a detailed epidemiological and serological survey in the city. Based on these observations, as a part of a wider study on leishmaniasis in Divinópolis, this work aimed to confirm the presence of *Leishmania infantum* in the city after 2009 and to assess the level of public awareness of the disease and aspects of its transmission.

## 2. Materials and Methods

### 2.1. Study Area and Data Collection

**Figure 1:**
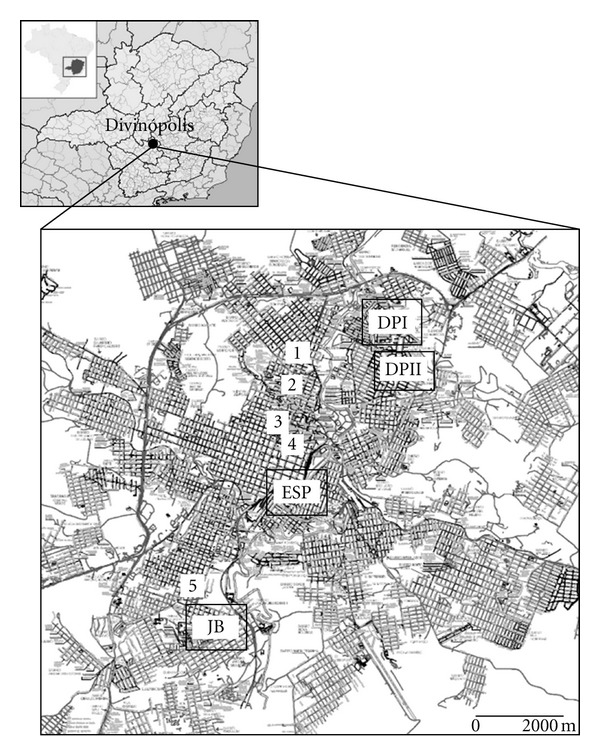
Divinópolis urban area. Rectangles indicate the four neighborhoods where questionnaires were applied, and numbers (1–5) indicate the five veterinary clinics surveyed. DPI, Danilo Passos I; DPII, Danilo Passos II; JB, Jardim Belvedere; ESP, Esplanada.

Divinópolis (20°8′21′′S, 44°53′17′′W) is located in west central Minas Gerais State, (Figure 1). Data on canine and human leishmaniasis (2004–2010) were obtained from the Reference Center of Epidemiological Surveillance (CREVISA) and the Epidemiology Department of City Hall (DEDCH), respectively [23, 24]. The project was approved by the Ethical Committee from FUNEDI/UEMG (protocol 63/2007) and FIOCRUZ (protocol P-0119-02).

### 2.2. Elaboration of Questionnaires and Distribution

 Four neighborhoods were evaluated (Figure 1): Esplanada and Jardim Belvedere, where the majority of human cases have occurred (1989–1991) (34) (52.5 and 32.5%, resp.), and Danilo Passos I and II (6) (12.5 and 2.5%, resp.). One hundred questionnaires (25 per neighborhood) were administered and sample size was calculated as described elsewhere [25]. The interviewed areas had similar characteristics. For example, only houses were present in the streets and buildings were absent. The questionnaires were based on previous VL studies (but also applicable to CL) and included general questions on sociodemographics, transmission, prevention, treatment, and environmental conditions [26]. All questionnaires were administered by the same person (M.E.O.), and informed consent was obtained. Selection of interview subjects was as follows: all streets in each neighborhood were numbered, and five were randomly selected. One home per block was randomly selected, totaling five houses per street and 25 homes per neighborhood. After selection of a given house, the next house would be in the subsequent block. The houses were always in the center of the block and never on a corner or at the end of the street.

### 2.3. Analyses

The percents of each response were calculated using Statistical Analysis System (SAS) software. Data were analyzed using the Kruskal-Wallis test. *P* < 0.05 was considered statistically significant.

### 2.4. Immunofluorescence and ELISA

 Sixty-nine dogs suspected of being infected with *Leishmania* from five veterinary clinics were subjected to serological tests (ELISA and RIFI) to detect *Leishmania *infection. Canine IFI-leishmaniasis and canine leishmaniasis EIA kits (Bio-Manguinhos/FIOCRUZ) were used according to the manufacturer's instructions for immunofluorescence and ELISA assays, respectively. Canine serum samples obtained from five veterinary clinics (Figure 1) were diluted from 1 : 40 (the cut-off value) to 1 : 640 in phosphate-buffered saline (PBS), and the cut-off value of absorbance was considered >0.05 OD. Data are a representation of two experiments in triplicate.

### 2.5. Restriction Fragment Length Polymorphism (RFLP-PCR)

Blood samples were subjected to DNA extraction for *Leishmania* detection [20, 22]. PCR reactions and thermal profile followed the procedure previously published [27, 28]. Amplified PCR products were digested with *HaeIII* (1 U, 3 h, 37°C) and visualized in polyacrylamide gels (8%). Data are a representation of two experiments.

## 3. Results

### 3.1. Social and Demographic Indicators

Females represented 71% percent of respondents in all neighborhoods, an indication that the majority of residents found at home were homemakers. In all studied areas, 69% of the population had completed high school and 63% of family incomes ranged from $300 to $900 (2008-2009). Education and income levels were similar among the four neighborhoods (*P* > 0.05).

### 3.2. Awareness of Leishmaniasis Transmission and Prevention

There was no difference among the four areas with respect to knowledge about leishmaniasis (*P* > 0.05). Approximately half of the respondents were unaware of the disease and its transmission routes and mechanisms (Table 2). Twenty-nine percent were aware that transmission occurred through the bite of the sand fly. In all interviewed individuals, dogs were identified as the main reservoir (49%), followed by rats (17%) and cats (4%) in all four areas (*P* = 0.0021). Thirty percent of the respondents did not know about reservoirs. In all neighborhoods, no difference was observed regarding prevention measures. Cleaning of yards and vacant lots was the most cited (38%), followed by dog euthanasia (17%). Thirty-three percent were not aware of any method of prevention. Most interviewees did not know about treatment (41%) and would take a suspected patient to a hospital (77%) or health agent (19%) (Table 2). Among the eight interviewees reporting previous leishmaniasis infection, four cases occurred in Esplanada (50%), two (25%) each in Jardim Belvedere and Danilo Passos II, and none in Danilo Passos I (data not shown), conforming to our prior information on incidence in the area [23, 24].

### 3.3. Environmental Conditions

In all surveyed neighborhoods, 60% of homes included pets, with a predominance of dogs (88.3%), followed by cats (3.3%) and other animals (8.3%) (*P* = 0.0346) (Table 3). No difference was observed in the number of dogs while comparing Danilo Passos I/II and Esplanada/Jardim (*P* > 0.05). The perception of the presence of hematophagous insects and rodents in the homes was reported in all neighborhoods (above 60%), with no observed difference among them (*P* > 0.05) (Table 3).

The majority of the surveyed homes in Jardim Belvedere (88%) and Danilo Passos II (72%) were near vacant lots. No difference among the four studied areas was observed with respect to proximity to water, green areas, presence of yard, and yard cleaning (Table 3). All homes had their trash regularly collected (data not shown).

### 3.4. Dog Survey

**Figure 2:**
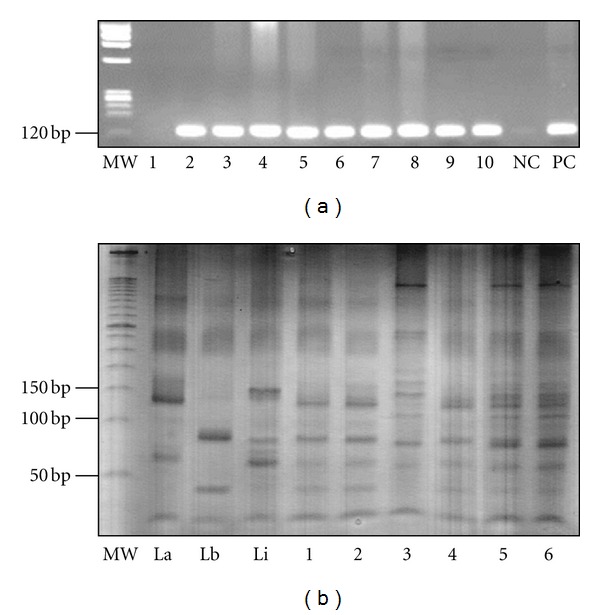
Molecular detection of *Leishmania infantum* from canine samples obtained in five veterinary clinics in Divinópolis, Brazil. (a) Detection of *Leishmania* sp. Legend: MW, molecular weight; lane 1, negative dog; lanes 2–10, positive dogs; NC, negative control; PC, positive control. (b) MW, molecular weight 50 bp ladder (Invitrogen, Carlsbad, CA, USA), lanes 1–3, positive controls represented by *Leishmania amazonensis*, *L. braziliensis* and *L. infantum*; Lanes 1–6, positive dogs for *L. infantum*.

Twenty-seven dogs (39.1%) tested positive using serological tests, with infection rates among the clinics varying from 6.25% to 50% (Table 4). Seventeen dogs (24.6%) were positive with both tests, and ten dogs (14.5%) were positive only with ELISA. For this reason, a more sensitive technique (PCR-RFLP) was conducted in those animals to confirm infection. A 120 bp fragment confirmed *Leishmania *sp. DNA in 100% of the blood samples. In the gel, PCR from nine blood samples is represented (lanes 2–10, Figure 2(a)). After digestion with *HaeIII*, *L. infantum* was confirmed as the species causing canine VL (lanes 1–6, Figure 2(b)).

## 4. Discussion

### 4.1. Urbanization as a Current Problem in Leishmaniases

Despite control programs, reports of leishmaniasis have been increasing. Many factors are involved, but it is clear that the lack of a vaccine, the adaptation of vectors and reservoirs to human environments, lack of effective drugs, and therapeutic failures contribute [29]. In Brazil, VL urbanization has been observed in places including São Luís, Natal, Teresina, Aracaju, Sobral, Boa Vista, Santarém, Cuiabá, Campo Grande, and Araçatuba [30–33]. In Minas Gerais state, VL urbanization has occurred in Montes Claros and Belo Horizonte [13, 30, 34]. It is not known if this phenomenon is occurring in the city of Divinópolis. Although human and canine cases have been reported, only serological and clinical diagnoses were made, with no parasitological investigation. Few studies have assessed the public perceptions of and attitudes toward the disease in this city.

### 4.2. Leishmaniasis Transmission, Prevention, and Environmental Conditions

 Analysis of the questionnaire administered in this study indicated a lack of knowledge of the disease. In all surveyed neighborhoods half of the interviewed subjects were unaware of the disease and aspects of its transmission. Similar situations have been reported in Belo Horizonte [35], Maringá [36], São Luís [26], and Tancredo Neves [37]. Similar results were also found in an area endemic for LTA in Venezuela, where 68% of the population had an insufficient level of information about transmission and prevention [38]. In our study, there was no difference in the level of knowledge about the risk factors and transmission of the disease. Forty-nine percent were able to identify the dog as a possible domestic reservoir. In a similar survey in São Luís, 87.2% of the respondents implicated the dog in leishmaniasis transmission [26].

The association of the vector with a domestic vertebrate host was not clear. Although 71% of the respondents reported the presence of hematophagous insects indoors, this does not indicate that they were sand flies. Sand flies are extremely small and difficult to identify compared to mosquitoes [39]. Only 29% of the house-holders knew that phlebotomine sand flies were responsible for leishmaniasis transmission. Similar results were observed in a transmission area in India, where VL is a major health problem, with 61% of the respondents believing mosquitoes to be the vectors of the disease. Currently there is no available information on sand fly species in the urban area of Divinópolis. Margonari et al. (2010) observed a high diversity of sand flies, including CL vectors, in Gafanhoto Park, a forest remnant in the city [22]. Consistent with those data, our questionnaires found some individuals that reported having been infected with CL in the 1990s, especially in the neighborhoods of Esplanada and Jardim Belvedere, strong evidence that the disease is occurring in town. The respondents were unclear as to the difference between the cutaneous and visceral forms of the disease.

Many reports have suggested that prevention measures face difficulties during implementation due to the lack of a public informed on basic concepts of the disease [26, 35, 37]. The large majority of the interviewed subjects were unaware of prevention measures and treatment, with the primary response being to take a victim to a hospital. In our survey, cleaning of yards and vacant lots was indicated as possible preventative measures. This probably reflects a common habit of the population rather than an action specific to prevention of leishmaniasis. Cleaning of yards is important to control vector proliferation [40] and synanthropic rodent occurrence. However, 45% of respondents reported the perception of the presence of those animals near their homes. In other urban and rural areas, some studies have incriminated them as *Leishmania* reservoirs, especially in the state of Pernambuco, Brazil [41, 42]. In a survey in the state of Minas Gerais, the presence of *L. mexicana*, *L. braziliensis*, and *L. donovani *complex species was detected in wild and synanthropic (*Rattus rattus*) rodents [43]. In Divinópolis, although rodents have been observed in wild areas (Gafanhoto Park), there was no parasite isolation/detection from those reservoirs. The presence of *L. braziliensis* and *L. infantum* was confirmed in this area after examination of sand flies [22]. More studies should be conducted to identify the role of wild and urban rodents as potential reservoirs for leishmaniasis in Divinópolis. There was no difference in the environmental aspects of the survey neighborhoods regarding proximity to forests or water bodies or collection areas. During our statistical analyses, we could not correlate their answers to any sociodemographic parameter. For this reason, a more detailed epidemiological analysis crossing those variables was not performed.

### 4.3. Dog Survey and Detection of Leishmania Infantum

 The domestic dog (*Canis familiaris*) is the main reservoir for VL and responsible for the endemic foci of leishmaniasis in urban and rural areas [3, 44]. In many transmission areas, a high incidence of human cases overlaps with high prevalence in canines [5, 45]. A recent study in Montes Claros, Minas Gerais, confirmed this using geo-referenced data identifying the main transmission areas in the city [46]. Since the dog was identified as the most common domestic reservoir by questionnaire respondents, our next step was to investigate the occurrence of canine VL in the city of Divinópolis. For this purpose, five strategically located veterinary clinics (Figure 1) selected animals suspected of being infected with *Leishmania* for the survey. The veterinaries knowledge was not assessed in this survey. After serological diagnosis, the presence of *Leishmania *sp. was detected. However, ELISA and RIFI did not identify the species involved. A more sensitive PCR-RFLP technique was performed that confirmed the presence of the parasite and identified *L. infantum *as the etiological agent of VL in Divinópolis. These data confirmed the parasite in the urban area and the dog as an important reservoir in the city. However, a more detailed epidemiological study is still warranted to describe incidence and prevalence.

## 5. Conclusions

This is the first study assessing public knowledge of several aspects of leishmaniasis in Divinópolis, Brazil, where many human cutaneous and visceral cases have been reported in the past two decades. Our data indicated that public knowledge is sparse and fragmented, suggesting the urgent need for leishmaniasis education and development of preventive methods. The study also demonstrated for the first time the occurrence of *L. infantum* in the canine population of the surveyed region, reflecting a possible disease urbanization process in recent years. 

## Figures and Tables

**Table 1:** Number of leishmaniasis cases reported (2004–2010) in Divinópolis according to regions.

Region	2004			2005			2006			2007			2008			2009			2010		
	CL	VL	CVL	CL	VL	CVL	CL	VL	CVL	CL	VL	CVL	CL	VL	CVL	CL	VL	CVL	CL	VL	CVL
Central^a^	—	—	0	—	—	2	—	—	2	—	—	1	—	**—**	13	—	—	32	2	1	39
Northeast^b^	1	—	1	—	—	0	—	—	1	—	—	1	1	—	0	—	1	13	5	2	63
Far Northeast	1	—	0	—	—	0	1	—	0	—	—	0	—	—	0	—	—	4	—	—	4
Northwest	—	—	0	—	—	3	—	—	0	1	—	0	—	—	2	—	—	15	2	—	32
Far Northwest	—	—	0	—	—	0	—	—	0	—	—	0	—	—	1	—	—	0	—	1	5
West	—	—	0	—	—	0	—	—	0	—	—	0	—	—	2	—	—	3	—	—	3
Southeast	—	—	0	—	—	0	—	—	0	—	—	1	—	—	1	1	—	19	2	1	47
Far Southeast	—	—	0	1	—	0	—	—	0	—	—	0	—	—	0	—	—	0	—	—	2
Southwest^d^	—	—	0	—	—	0	—	—	0	—	—	1	—	—	1	—	—	14	3	—	14
Far Southwest	—	—	0	—	—	0	—	—	0	1	—	0	—	—	0	—	—	0	2	—	6

Total	2	0	1	1	0	5	1	0	3	2	0	4	1	0	20	1	1	100	16	5	215

^a^Region of Esplanada (see Figure 2 for details); ^b^region of Danilo Passos I and II (see Figure 2 for details); ^c^region of Jardim Belvedere (see Figure 2 for details); CL: human cutaneous leishmaniasis; VL: human visceral leishmaniasis and CVL: canine visceral leishmaniasis. Data obtained from CREVISA [24].

**Table 2:** Frequency of responses regarding leishmaniasis knowledge in four neighborhoods of Divinópolis, MG, Brazil.

Question	Number (%)			
	Neighborhood			
	DPI	DPII	ESP	BEL
Do you know what leishmaniasis is?*				
Yes	13 (52)	10 (40)	13 (52)	13 (52)
No	12 (48)	15 (60)	12 (48)	12 (48)
Do you know how it is transmitted?*				
Do not know	13 (52)	13 (52)	13 (52)	13 (52)
Sand fly	7 (28)	8 (32)	7 (28)	7 (28)
Dog	4 (16)	4 (16)	4 (16)	5 (20)
Other	1 (4)	0 (0)	1 (4)	0 (0)
Do you know the reservoir?*				
Dog	10 (40)	11 (44)	12 (48)	16 (64)
Rat	6 (24)	3 (12)	4 (16)	4 (16)
Cat	1 (4)	2 (8)	0 (0)	1 (4)
Do not know	8 (32)	9 (36)	9 (36)	4 (16)
Do you know how to prevent?*				
Do not know	8 (32)	10 (40)	10 (40)	5 (20)
Yard cleaning	9 (36)	11 (44)	7 (28)	11 (44)
Dog euthanasia	4 (16)	2 (8)	6 (24)	5 (20)
Water accumulation	1 (4)	2 (8)	0 (0)	3 (12)
Insecticides	2 (8)	0 (0)	1 (4)	1 (4)
Other	1 (4)	0 (0)	1 (4)	0 (0)
What would you do to help a suspected victim?				
Take to hospital	19 (76)	21 (84)	18 (72)	19 (76)
Take to a health agent	6 (24)	3 (12)	4 (16)	6 (24)
Do not know	0 (0)	1 (4)	1 (4)	0 (0)
How would you treat leishmaniasis?**				
No treatment	11 (44)	8 (32)	10 (40)	4 (16)
Glucantime	2 (8)	0 (0)	1 (4)	1 (4)
Antibiotics	0 (0)	2 (8)	1 (4)	1 (4)
Vaccine	1 (4)	1 (4)	0 (0)	4 (16)
Do not know	8 (32)	12 (48)	11 (44)	10 (40)
Other	3 (12)	2 (8)	2 (8)	5 (20)

DPI: Danilo Passos I; DPII: Danilo Passos II; ESP: Esplanada; BEL: Belvedere. *No statistical difference was observed among the four neighborhoods (Kruskall-Wallis, *P* > 0.05). **No treatment in this case means that they are not aware that leishmaniasis has a treatment for humans.

**Table 3:** Frequency of responses regarding transmission risk of leishmaniasis in four neighborhoods of Divinópolis, MG, Brazil.

Question	Number (%)			
	Neighborhood			
	DPI	DPII	ESP	BEL
Do you have pets?				
Dog	14 (93)	14 (93)	15 (79)	10 (90)
Cat	0 (0)	1 (7)	1 (5)	0 (0)
Other	1 (7)	0 (0)	3 (16)	1 (10)
Did you notice blood-sucking insects in the house?*				
Yes	18 (72)	20 (80)	18 (72)	15 (60)
No	7 (28)	5 (20)	7 (28)	10 (40)
Did you notice rodents around the home area?*				
Yes	14 (56)	16 (64)	5 (20)	10 (40)
No	11 (44)	9 (36)	20 (80)	15 (60)*
Are there any vacant lots in the surroundings?				
Yes	3 (12)	18 (72)	2 (8)	22 (88)
No	22 (88)	7 (28)	23 (92)	3 (12)
Is there any water collection/river close to the house?				
Yes	24 (96)	19 (76)	16 (64)	20 (80)
No	1 (4)	6 (24)	9 (36)	5 (20)
Is there any green area close to the house?				
Yes	18 (72)	25 (100)	8 (32)	24 (96)
No	7 (28)	0 (0)	17 (68)	1 (4)
Is there any backyard at home?				
Yes	14 (56)	16 (54)	18 (72)	15 (60)
No	11 (44)	9 (36)	7 (28)	10 (40)
Do you clean your backyard regularly?				
Yes	24 (96)	23 (92)	21 (84)	24 (96)
No	1 (4)	2 (8)	3 (12)	1 (4)

DPI: Danilo Passos I; DPII: Danilo Passos II; ESP: Esplanada; BEL: Belvedere. *Based on population perception and not sampling.

**Table 4:** Proportion of dogs from five veterinary clinics in Divinópolis, MG, Brazil, positive for leishmaniasis with serology tests (ELISA/RIFI) and PCR-RFLP.

Clinic	Samples *n*	Serology *n* (%)
1	10	5 (50)
2	16	1 (6.25)
3	24	12 (50)
4	19	8 (42)
5	2	1 (50)
